# Is t(11;14) in newly diagnosed multiple myeloma a favorable outcome in the novel agent era?

**DOI:** 10.1007/s44313-025-00056-8

**Published:** 2025-02-06

**Authors:** Ye Li, Jingjing Deng, Yuan Jian, Zhiyao Zhang, Wenming Chen

**Affiliations:** 1 Peking University People’s Hospital, Peking University Institute of Hematology, National Clinical Research Center for Hematologic Disease, Beijing Key Laboratory of Hematopoietic Stem Cell Transplantation, Beijing, China 100044; 2 Department of Hematology, Myeloma Research Center of Beijing, Beijing Chaoyang Hospital, Capital Medical University, Beijing, China 100020

**Keywords:** T(11;14), Multiple Myeloma, 1q21 +, High-risk cytogenetic abnormalities, Del(17p)

## Abstract

**Background:**

t(11;14) is considered a standard risk factor in multiple myeloma (MM). However, recent studies suggested that its impact in the context of novel agents remained controversial.

**Methods:**

This retrospective analysis examined the clinical profiles of 375 newly diagnosed patients with MM and compared the outcomes between those with t(11;14) and those with normal cytogenetics.

**Results:**

The median progression-free survival (PFS) of the 84 patients with t(11;14) was 36 months (95% confidence interval (CI), 23.5–48.5), which was significantly shorter than the median PFS of 65 months (95% CI, 23.0–107.0) for the 59 patients with normal cytogenetics (*p* = 0.011). Median overall survival (OS) was not reached in either group (*p* = 0.977). When combined with 1q21 + , t(11;14) showed a trend toward poorer PFS (median PFS: 36 vs. 65 months; *p* = 0.130). In the presence of high-risk cytogenetics (HRCAs), t(11;14) was associated with a worse PFS (median PFS: 9 vs. 38 months, *p* = 0.015) and a trend toward shorter OS (median OS: 33 vs. 49 months, *p* = 0.096). Multivariate analysis indicated that t(11;14) was a poor prognostic factor for PFS. 1q21 + was a detrimental prognostic factor, particularly in the t(11;14) group. Autologous stem cell transplantation (ASCT) may be a beneficial treatment option for patients with t(11;14).

**Conclusion:**

In this study, patients with MM with t(11;14) demonstrated poorer PFS than those with normal cytogenetics. Further investigations are required to evaluate the impact of t(11;14) in patients newly diagnosed with MM in the era of novel agents.

## Introduction

Multiple myeloma (MM) is a genetically heterogeneous and incurable hematological malignancy [1], and variants caused by diverse genomic abnormalities significantly influence patient outcomes [2]. Primary genetic alterations, including hyperdiploidy and translocations involving the immunoglobulin heavy chain (IgH) enhancer on chromosome 14, are particularly relevant [3]. *Fluorescence *in situ* hybridization* (FISH) remains the standard method for risk stratification in MM [4, 5]. IGH translocations encompass both high-risk cytogenetic aberrations (HRCAs), such as t(4;14), t(14;16), and t(14;20) [6, 7], and standard-risk translocations, such as t(11;14) and t(6;14). The t(11;14) translocation, observed in 15–20% of MM case [8–10], has historically been classified as a standard-risk chromosomal abnormality in the pre-novel agent era [5, 11]. However, there have been conflicting reports regarding its prognostic implications [12, 13]. Patients with t(11;14) reportedly had diminished sensitivity to bortezomib, along with significantly lower deep response rates and progression-free survival (PFS), compared to standard-risk groups [8]. A recent study indicated that patients with t(11;14) may experience worse outcomes than those with standard-risk MM [14], whereas another study suggested that the prognosis of t(11;14) MM aligns more closely with that of a standard-risk cohort [15]. Most previous studies are retrospective, introducing inherent biases and have reported variable incidences of HRCAs, potentially contributing to discrepancies in outcomes.

The t(11;14) translocation frequently occurs alongside other abnormalities, with approximately one-third of affected patients exhibiting additional cytogenetic abnormalities (CAs) [15–19]. Recent studies highlight the need to consider the presence of coexisting CAs when assessing the prognostic implications of t(11;14) [20, 21]. Several reports suggest that secondary abnormalities, such as del(17p) or 1q21 + , may significantly affect the clinical outcomes of patients with t(11;14) [17, 18, 22–24]. However, few studies have explored the influence of t(11;14) on CAs.

A comprehensive analysis was conducted on 375 patients with newly diagnosed multiple myeloma (NDMM) from a single center in China to assess the survival outcomes of those with t(11;14) within the context of novel therapeutic agents and to evaluate its interaction with concurrent CAs. This study provides an in-depth exploration of genetic heterogeneity in clinical practice, shedding light on complex genetic landscapes observed in real-world settings.

## Patients and methods

### Patients and methods

A retrospective analysis was conducted on patients with NDMM treated at the Beijing Chaoyang Hospital between January 2018 and September 2020. Among the 375 patients who met the inclusion criteria and underwent pretreatment FISH, 84 presented with t(11;14). Baseline data encompassed parameters such as age, hemoglobin, creatinine, calcium, albumin, lactate dehydrogenase (LDH), β2-microglobulin (β2MG), isotype, International Staging System (ISS) stage, and initial treatment. All patients were diagnosed based on the International Myeloma Working Group (IMWG) MM criteria [25] and monitored until September 1, 2023.

Induction regimens incorporating at least one novel agent, such as proteasome inhibitor (PI)-based, immunomodulatory drug (IMID)-based, or combined PI-IMID therapies, were administered to all patients. For those eligible for transplantation, a four-course induction phase preceded autologous hematopoietic stem cell transplantation (ASCT) as a consolidation therapy. Three months post-ASCT, maintenance therapy was initiated with medications such as lenalidomide and treatment efficacy was evaluated following the IMWG response criteria.

Samples were isolated from the bone marrow using CD138 microspheres and subjected to FISH analysis with DNA probes specific to chromosomal abnormalities, including *TP53(17p13.1), 1q21(1q21), IGH/MAF (14q32/16q23), IGH/FGFR3(14q32/4p16.3),* and *IGH/CCND1(14q32/11q13)*. The analysis covered 200 interphase nuclei, and probe cutoff values were defined as 6.09% for del(17p), 6.87% for 1q21 + , 4.85% for t(11;14), 6.47% for t(4;14), and 3.37% for t(14;16).

### Statistical analysis

Overall survival (OS) was defined as the duration from diagnosis until death or the most recent follow-up. PFS denoted the time span from the commencement of treatment to disease progression, recurrence, or mortality due to any cause.

The clinical characteristics of continuous variables are summarized as medians and ranges, while categorical covariates are expressed as frequencies and percentages. Comparisons between categorical variables across groups were conducted using Fisher’s exact test, and the Mann–Whitney U test was used for continuous variables. Kaplan–Meier survival curves were generated, and differences were assessed using the log-rank test. Univariate Cox regression analysis was used to assess the effects of baseline variables, and multivariate analysis of PFS and OS was performed using a Cox proportional hazards model, which also provided hazard ratios (HR) and 95% confidence intervals (CI). All statistical analyses were performed using SPSS (version 29.0), GraphPad Prism 9 (GraphPad Software Inc., La Jolla, CA, USA) and R 4.3.1 (R Foundation for Statistical Computing, Vienna, Austria), with statistical significance defined as *p* < 0.05.

## Result

### Baseline characteristics of patients

Table 1 presents the baseline characteristics and survival outcomes of the entire cohort in the t(11;14) (*n* = 84) and normal cytogenetics groups (*n* = 59). No significant differences in baseline characteristics were observed between the two groups, except for creatinine levels. The induction and maintenance treatment plans for 84 patients with t(11;14) and 59 normal cytogenetics cases are described in the Supplementary Material.

**Table 1:** Baseline characteristics of 84 cases in the t(11; 14) group and 59 cases in the normal cytogenetics group

Variable	Normal cytogenetics (*n* = 59)	t(11;14) (*n* = 84)	*p*-value
Age, median (range)	62 (33–82)	60 (33–84)	0.527
ISS stage, *n* (%)			0.066
1	19 (32.2)	15 (17.9)	
2	15 (25.4)	18 (21.4)	
3	25 (42.4)	51 (60.7)	
M-protein isotype, *n* (%)			0.277
IgG	27 (45.8)	27 (32.1)	
IgA	10 (16.9)	12 (14.3)	
IgD	5 (8.5)	8 (9.5)	
IgM	0	1 (1.2)	
κ	7 (11.9)	23 (27.4)	
λ	6 (10.2)	10 (11.9)	
Non-secretory	4 (6.8)	3 (3.6)	
Hemoglobin (g/L) < 100 g/L	25 (44.6)	46 (56.1)	0.226
LDH (U/L) ≥ ULN	8 (13.6)	12 (14.3)	1.000
Albumin (g/L), median (range)	37.5 (19.6–129.0)	39.5 (19–127)	0.403
Creatine (μmol/L), median (range)	70 (38.7–1161)	82.3 (36.7–1436)	0.049
Calcium (mmol/L) ≥ 2.75	3 (5.7)	14 (17.7)	0.062
β2-microglobulin (mg/L) ≥ 5.5	22 (41.5)	43 (56.6)	0.109
Induction, *n* (%)			0.177
PIs or IMIDs	29 (49.2)	51 (60.7)	
PIs + IMIDs	30 (51.8)	33 (39.3)	
ASCT, *n* (%)	24 (40.7)	25 (29.8)	0.211
Accompanying cytogenetics, *n* (%)			
1q21 +		30 (35.7)	
del(17p)		6 (7.14)	
t(4;14)		1 (1.19)	
t(14;16)		1 (1.19)	

*ASCT* autologous peripheral stem cell transplantation; *ISS* international staging system; *PI* proteasome inhibitors; *IMIDs* immunomodulatory drugs; *LDH* lactate dehydrogenase; *ULN* upper limit of normal

In the t(11;14) cohort, the median age was 62 years (range, 33–82), and males comprised 58.33% (49/84) of the population. Patients classified as ISS stages I, II, and III numbered 15, 18, and 51, respectively. The free light chain subtype of the M protein was detected in 39.28% (33/84) of patients. In the t(11;14) group, 47 patients presented with t(11;14) alone, while additional CAs were documented in 7 patients with combined HRCA [5 cases with del(17p), 1 case with del (17p) and t(14;16), 1 case with t(4;14)], and 1q21 + was identified in 34 patients.

### Survival

**Fig. 1:**
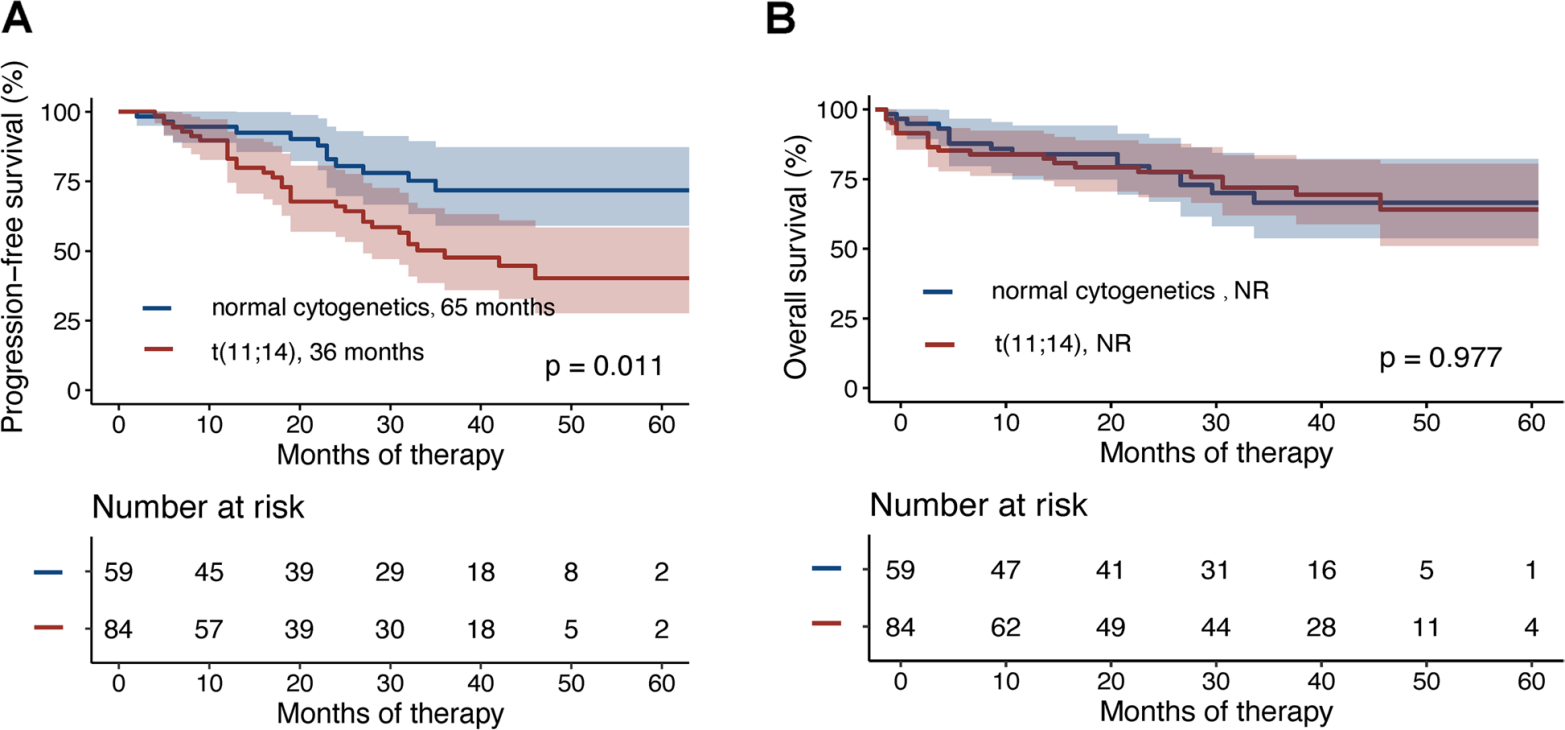
Kaplan–Meier analysis of 84 patients with t (11;14) and 59 normal cytogenetics cases for PFS (**A**) and OS (**B**)

The median follow-up duration for the 84 patients with t(11;14) was 32 months (range, 1–66 months). The median PFS was 36 months (95% CI, 23.5–48.5) for the t(11;14) group and 65 months (95% CI, 23.0–107.0) for the normal cytogenetics group (*p* = 0.011; Fig. 1A). The 3-year PFS rates were 47.7% (95% CI, 33.84–60.31%) for the t (11;14) group and 71.81% (95% CI, 55.08–83.21%) for the normal cytogenetics group. Median OS was not reached (NR) in either cohort (*p* = 0.977; Fig. 1B). The 3-year OS rates were 72.0% (95% CI, 59.63–81.16%) for the t(11;14) group and 66.51% (95% CI, 50.36–78.47%) for the normal cytogenetics group.

**Fig. 2:**
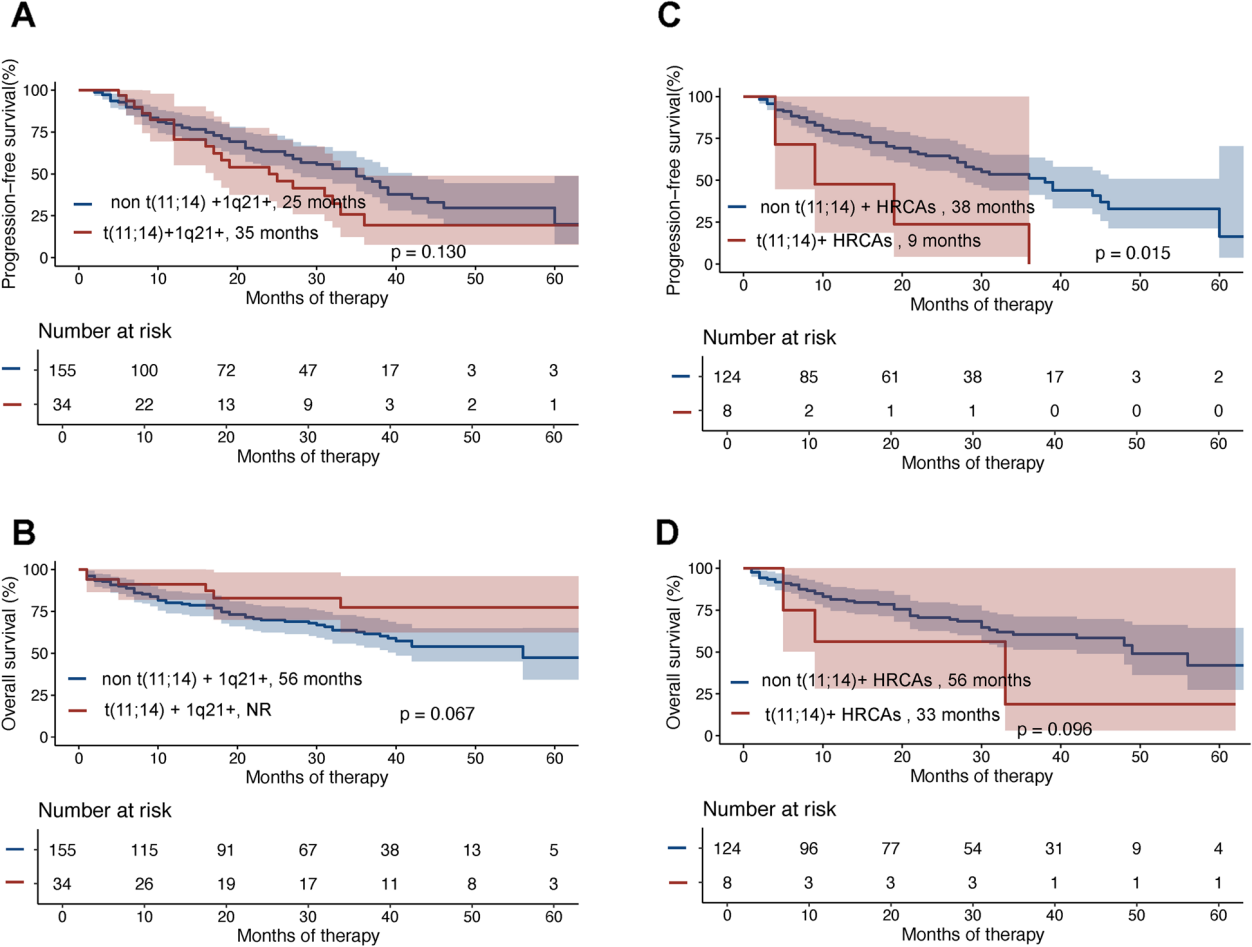
Kaplan–Meier analysis of t(11;14) + 1q21 + and non-t(11;14) + 1q21 + groups for PFS (**A**) and OS (**B**); analysis of t(11;14) + HRCAs and non-t(11;14) + HRCAs groups for PFS (**C**) and OS (**D**)

### The additional prognostic impact of t(11;14) in HRCA

To evaluate the additional impact of t(11;14) in HRCAs or 1q21 + , we compared patients with HRCAs or 1q21 + who do not company with t(11;14).

The median PFS was 25 months (95% CI, 13.41–34.60) and 35 months (95% CI, 29.17–40.83) for the 34 patients with 1q21 + combined with t(11;14) and 155 patients with 1q21 + combined with non-t(11;14), respectively (*p* = 0.130; Fig. 2A), while the 3-year PFS rates were 20.07% (95% CI, 5.79–40.44%) and 47.09% (95% CI, 36.69–56.81%), respectively. The median OS was 56 months and NR for the 1q21 + combined with t(11;14) group and 1q21 + combined with non-t(11;14) group, respectively (*p* = 0.067; Fig. 2B), while the 3-year OS rates were 61.54% (95% CI, 52.24–69.56%) and 77.39% (95% CI, 55.17–89.55%), respectively.

The median PFS was 9 months (95% CI, 0–22.91) and 38 months (95% CI, 28.29–47.21) for the 8 patients with HRCAs combined with t(11;14) and 124 patients with HRCAs combined with non-t(11;14), respectively (*p* = 0.015; Fig. 2C), while the 3-year PFS rates were 0 and 51.27% (95% CI, 39.84–61.56%) for the two groups, respectively. The median OS was 33 months (95% CI, 12.07–53.93) and 56 months (95% CI, 36.42–61.58) for the 8 patients with HRCAs combined with t(11;14) and 124 patients with HRCAs combined with non-t(11;14), respectively (*p* = 0.096; Fig. 2D), while the 3-year OS rates were 18.75% (95% CI, 0.87–55.40%) and 60.42% (95% CI, 49.88–69.41%) for the two groups, respectively.

### Analysis of prognostic factors of t(11;14)

Univariate and multivariate analyses were conducted to assess the effects of clinical parameters on PFS and OS in the 375 patients with NDMM. While univariate analysis indicated no statistical significance for t(11;14) regarding PFS (*p* = 0.434) or OS (*p* = 0.578), multivariate analysis revealed a significant negative prognostic effect of t(11;14) on PFS (*p* = 0.048; 95% CI, 1.004–2.497), but no significant association for OS (*p* = 0.968; 95% CI 0.610–1.673) (Table 2).

**Table 2:** Univariate and multivariate analysis of variables associated with outcomes in 375 patients with NDMM

	Univariate		Multivariate	
Variants	*p-*value	HR (95% CI)	*p*-value	HR (95% CI)
PFS				
t(11;14)	0.434	1.178 (0.782–1.774)	0.048	1.583 (1.004–2.497)
del(17p)	0.015	1.793 (1.119–2.872)	0.025	1.728 (1.070–2.790)
t(14;16)	< 0.001	4.427 (2.217–8.841)	< 0.001	3.984 (1.930–8.222)
t(4;14)	0.072	1.439 (0.968–2.14)	0.271	1.281 (0.824–1.992)
1q21 +	< 0.001	2.835 (1.953–4.115)	< .001	2.501 (1.704–3.671)
ISSII/III	0.018	1.766 (1.102–2.832)	0.251	1.331 (0.817–2.167)
LDH high	< 0.001	2.137 (1.417–3.224)	0.002	1.961 (1.279–3.006)
PIs + IMIDs	0.440	1.074 (0.895–1.289)	0.282	1.109 (0.919–1.339)
ASCT	0.643	0.918 (0.64–1.317)	0.607	0.907 (0.625–1.316)
OS				
t(11;14)	0.578	0.873 (0.542–1.407)	0.968	1.01 (0.610–1.673)
del(17p)	0.145	1.471 (0.876–2.47)	0.064	1.647 (0.971–2.793)
t(14;16)	0.018	2.397 (1.165–4.934)	0.036	2.230 (1.053–4.722)
t(4;14)	0.107	1.426 (0.927–2.193)	0.123	1.441 (0.906–2.292)
1q21 +	0.032	1.52 (1.038–2.227)	0.260	1.253 (0.846–1.857)
ISSII/III	< 0.001	3.346 (1.688–6.633)	0.008	2.568 (1.278–5.162)
LDH high	< 0.001	2.184 (1.433–3.33)	0.002	1.952 (1.272–2.996)
PIs + IMIDs	0.008	0.771 (0.636–0.933)	0.005	0.757 (0.622–0.920)
ASCT	< 0.001	0.337 (0.205–0.555)	< 0.001	0.339 (0.204–0.562)

*ASCT* autologous peripheral stem cell transplantation; *ISS* international staging system; *PI* proteasome inhibitors; *IMIDs* immunomodulatory drugs; *LDH* lactate dehydrogenase; *HR* hazards ratio; *CI* confidence interval; *PFS* progression-free survival; *OS* overall survival

Univariate and multivariate analyses were performed to explore the clinical parameters of PFS and OS in the 84 NDMM patients with t(11;14). Univariate analyses showed that 1q21 + (*p* = 0.007; 95% CI, 1.319–5.951), del(17p) (*p* = 0.049; 95% CI, 1.007–11.168) and bone marrow plasma-cell (BMPC) ≥ 50% (*p* = 0.033; 95% CI, 1.069–4.880) were risk factors affecting PFS, while ISS stage III (*p* = 0.007; 95% CI, 1.757–32.495), β2MG ≥ 5.5 mg/L (*p* = 0.029; 95% CI, 1.119–8.398), ASCT (*p* = 0.016; 95% CI, 0.011–0.636), BMPC ≥ 50% (*p* < 0.001; 95% CI, 2.515–22.350), and age ≥ 65 years (*p* = 0.013; 95% CI, 0.146–0.797) were risk factors affecting OS. Multivariate analysis showed that β2MG ≥ 5.5 mg/L (*p* = 0.008; 95% CI, 1.592–22.218), BMPC ≥ 50% (*p* = 0.022; 95% CI, 1.223–13.902), and 1q21+ (*p* < 0.001; 95% CI, 2.271–19.916) were independent prognostic factors affecting PFS, while ASCT (*p* = 0.021; 95% CI, 0.007–0.668) was an independent prognostic factor affecting OS (Table 3).

**Table 3:** Univariate and multivariate analysis of variables associated with outcomes in 84 patients with MM with t (11;14)

	Univariate		Multivariate	
Variants	*p*-value	HR (95% CI)	*p*-value	HR (95% CI)
PFS				
ISS III	0.143	1.756 (0.827–3.728)	0.122	0.302 (0.066–1.375)
Ca ≥ 2.75 mmol/L	0.407	0.682 (0.276–1.684)	0.093	0.364 (0.112–1.183)
β2MG ≥ 5.5 mg/L	0.056	2.081 (0.980–4.421)	0.008	5.948 (1.592–22.218)
ASCT	0.959	1.020 (0.488–2.132)	0.056	0.365 (0.13–1.025)
CD20	0.117	0.424 (0.145–1.238)	0.356	0.550 (0.154–1.960)
1q21 +	0.007	2.802 (1.319–5.951)	< 0.001	6.725 (2.271–19.916)
BMPC ≥ 50%	0.033	2.284 (1.069–4.880)	0.022	4.123 (1.223–13.902)
Age ≥ 65 years	0.364	1.477 (0.636–3.432)	0.785	1.172 (0.374–3.667)
del(17p)	0.049	3.353 (1.007–11.168)	0.053	4.748 (0.982–22.965)
PIs + IMIDs	0.569	1.224 (0.610–2.455)	0.270	1.918 (0.602–6.109)
OS				
ISS III	0.007	7.556 (1.757–32.495)	0.081	9.068 (0.763–107.809)
Ca ≥ 2.75 mmol/L	0.113	0.461 (0.177–1.201)	0.105	0.405 (0.136–1.207)
β2MG ≥ 5.5 mg/L	0.029	3.066 (1.119–8.398)	0.207	0.397 (0.095–1.667)
ASCT	0.016	0.085 (0.011–0.636)	0.021	0.070 (0.007–0.668)
CD20	0.453	0.658 (0.221–1.960)	0.492	0.63 (0.169–2.352)
1q21 +	0.231	0.561 (0.217–1.446)	0.341	0.603 (0.213–1.71)
BMPC ≥ 50%	< 0.001	7.498 (2.515–22.350)	0.197	2.365 (0.639–8.754)
Age ≥ 65 years	0.013	0.342 (0.146–0.797)	0.516	0.703 (0.243–2.036)
del(17p)	0.169	2.362 (0.694–8.036)	0.258	2.486 (0.513–12.053)
PIs + IMIDs	0.171	0.518 (0.202–1.328)	0.773	1.184 (0.377–3.716)

*BMPC* bone marrow plasma cells; *ISS* International Staging System; *β2MG* serum β2-microglobulin; *ASCT* autologous peripheral stem cell transplantation; *PIs* proteasome inhibitors; *IMIDs* immunomodulatory drugs; *LDH* lactate dehydrogenase; *HR* hazards ratio; *CI* confidence interval; *PFS* progression-free survival; *OS* overall survival

## Discussion

In this study, we found that patients with NDMM harboring t(11;14) had reduced PFS but no significant variation in OS compared to their standard-risk counterparts with normal cytogenetics, as determined by FISH. Notably, those with t(11;14) combined with other CAs, including 1q21 + or other HRCAs, demonstrated marginally lower survival rates than individuals with non-t(11;14).

This retrospective analysis of 375 patients with NDMM identified a t(11;14) prevalence of 22.4%, consistent with previous reports [9, 10, 15, 26]. As a distinct subgroup, t(11;14) MM exhibits unique biological and clinical features, characterized by lymphoid plasma cell morphology, elevated circulating plasma cells, CD20 expression in tumor plasma cells, IgG λ subtype, and both low-secretion and non-secretion phenotypes [8–10, 15, 22, 27, 28]. Table 1 outlined the clinical details of 84 t(11;14) patients, with a median age of 62 years (33-82), the majority being male. The cohort comprised 39.28% light chain type, 3 cases of IgD (3.57%), and 3 cases of the non-secretory type (3.57%), consistent with previous findings.

In recent years, the introduction of novel targeted agents, such as IMIDs and PIs, has improved the OS of patients with MM. However, those with t(11;14) did not experience similar benefits. The prognostic impact of t(11;14) in patients with NDMM has been re-examined in recent studies. Lakshman et al. conducted a comparative analysis of PFS and OS between patients with t(11;14) (n = 365) and matched controls (n = 730), revealing significantly shorter PFS and OS in the t(11;14) cohort compared to those without the translocation [26]. A 10-year follow-up by the Australian Lymphoma Leukaemia Group, involving 74 t(11;14) patients, further indicated that t(11;14) may be associated with a less favorable risk profile [29]. Additionally, Gran et al. reviewed 469 patients with NDMM and demonstrated that t(11;14) was correlated with poorer outcomes, particularly in standard-risk patients not treated with high-dose therapy, with a significantly shorter PFS observed in the t(11;14) standard-risk group compared to the non-t(11;14) standard-risk group (*p* = 0.01) [30]. A multicenter study in South Korea, involving 290 cases, identified t(11;14) as a poor prognostic factor in patients with MM with extramedullary plasmacytoma undergoing ASCT (HR = 25.154, *p* < 0.001 for PFS; HR = 7.484, *p* = 0.024 for OS) [31]. Similarly, in our cohort, analysis revealed that the 84 patients with t(11;14) had a significantly shorter PFS (median PFS: 36 months vs. 65 months; *p* = 0.011) but similar OS (median OS: NR vs. NR; *p* = 0.977) compared to the 59 patients with normal cytogenetics. However, other studies have reported that patients with t(11;14) exhibit outcomes similar to those with standard risk when treated with novel agents. In a study by Liu Yang, diagnosis time was used as the matching variable, and 109 non-t(11;14) patients with NDMM were randomly selected as the control group in a 1:1 ratio for comparison. The results indicated a trend toward longer OS in the t(11;14) group compared to the non-t(11;14) group (7.25 years vs. 4.75 years, *p* = 0.074) [17]. This discrepancy may be attributed to the higher prevalence of gain/amp 1q21 in the non-t(11;14) group compared to the t(11;14) cohort (60.6% vs. 45.9%, *p* = 0.026), which could influence survival outcomes in the non-t(11;14) group. A multicenter study conducted in China evaluated 455 patients with MM who underwent ASCT as consolidation following induction therapy. The analysis revealed no significant differences in PFS and OS between the t(11;14) and standard risk groups, with a median PFS of 52 vs. 63 months (*p* = 0.935) and median OS of 86 vs. 100 months (*p* = 0.836) [15]. While some patients in this cohort received induction therapy with conventional agents, all patients received ASCT as consolidation therapy. Similarly, a propensity-score matched analysis examining the effect of ASCT in patients with MM with t(11;14) demonstrated comparable outcomes for PFS (median PFS: 29.9 vs. 51.9 months; *p* = 0.140) and OS (median OS: NR vs. NR; *p* = 0.170) between 80 t(11;14) patients and 80 standard-risk patients [23].

This study indicates that ASCT may mitigate the poor prognosis associated with t(11;14) in patients with MM. A study from Japan involving 97 patients with MM with t(11;14) who underwent a single ASCT, reported no significant differences in PFS and OS between patients with MM with t(11;14) and those with normal cytogenetics [22]. Similarly, a single-center study in China demonstrated that despite the presence of 1q21 + , those with t(11;14) who received ASCT showed a tendency toward improved OS compared to those who did not undergo ASCT (*p* = 0.076) [17]. Although ASCT appears to improve the survival rates of patients with t(11;14), treatment in resource-limited countries continues to pose challenges, highlighting the need for novel therapeutic approaches to enhance patient outcomes.

A notable feature of t(11;14) MM is the elevated expression of the anti-apoptotic protein B-cell lymphoma 2 (BCL-2) in clonal plasma cells, whereas the proapoptotic proteins myeloid cell leukemia-1 (MCL-1) and BCL-XL are expressed at lower levels, rendering the cells more sensitive to BCL-2 inhibitors [32]. A study evaluating venetoclax combined with daratumumab and dexamethasone (VenDd) and VenDd with bortezomib (VenDVd) in relapsed or refractory MM (RRMM) with t(11;14) demonstrated a marked improvement in overall response rates. The 18-month PFS rates were 90.5% for VenDd and 66.7% for VenDVd across 24 cases in each group [33]. Further investigation of venetoclax in patients with RRMM with t(11;14) revealed a strong response. In a cohort of 66 patients, 86% (12/14) of those with favorable responses had t(11;14) and 27% achieved very good partial response (VGPR) [34]. Although the recommendation of venetoclax for patients with NDMM with t(11;14) remains uncertain, anti-BCL-2 agents have demonstrated notable efficacy in this subgroup. Thus, exploring anti-BCL-2 targeted therapies in the early stages of MM treatment is a promising approach.

Research has demonstrated that the presence of multiple HRCAs in the same patient has a more profound impact on prognosis than isolated CAs [20]. In the Myeloma IX study, Pawlyn et al. analyzed 127 patients with t(11;14) and identified del(17p) or 1q21 + as independent risk factors for OS (*p* = 0.050), with a non-significant trend observed for PFS (*p* = 0.108) [19]. Further analysis confirmed that the addition of specific CAs to t(11;14) is associated with worse outcomes, placing patients with co-occurring adverse lesions in the high-risk category [24]. Similarly, Saini et al. reported that t(11;14) accompanied by either high-risk or non-high-risk CAs leads to poor prognosis, indicating that t(11;14) does not negate poor outcomes associated with high-risk cytogenetics [23]. Takamatsu et al. found that among 97 patients with MM who underwent single planned ASCT, the median OS in the t(11;14) group with additional CAs (46.2 months) was significantly shorter than that in the group without additional CAs (NR) (*p* = 0.005) [22]. Evidence further suggests that the co-occurrence of 1q21 + correlates with worse prognosis and that 1q21 + is an independent prognostic factor for both PFS and OS [17]. However, a Chinese multicenter study suggested that among patients receiving ASCT, t(11;14) patients, regardless of 1q21 status, exhibited similar median PFS and OS as those in the standard-risk group.

This study has certain limitations. First, this was a single-center retrospective study, which may have introduced bias. Second, the sample size was relatively small and larger studies are needed to further validate the prognosis of t(11;14) in the era of new drugs. The heterogeneity in treatment outcomes between patients with t(11;14) alterations and individuals with normal cytogenetics may affect PFS in both the groups. Finally, the follow-up period was relatively short.

In summary, our research indicates that in the era of new drugs, patients with t(11;14) have poorer PFS compared to those with normal cytogenetics. Moreover, the presence of additional CAs, such as HRCAs and 1q21 + , correlates with an even worse prognosis. Notably, 1q21 + is considered a prognostic risk factor for PFS in t(11;14) MM. Consequently, alternative therapeutic options are essential for clinical treatment of patients with NDMM with this genetic abnormality.

## Data Availability

No datasets were generated or analysed during the current study.
